# Armed conflict exposure types are not equally associated with access to psychosocial support: A study of over 8 million victims of the Colombian armed conflict

**DOI:** 10.1177/00207640251336726

**Published:** 2025-05-27

**Authors:** Charlotte Constable Fernandez, Alida Acosta-Ortiz, María Camila García Durán, Rob Saunders, Francesca Solmi, William Tamayo-Agudelo, Fabio Idrobo, Vaughan Bell

**Affiliations:** 1 Clinical, Educational and Health Psychology, University College London, UK; 2 Universidad Autónoma de Bucaramanga, Santander, Colombia; 3 Salud Poblacional, Fundación Santa Fe de Bogotá, Colombia; 4 Department of Health Services and Population Research, King’s College London, UK; 5 Division of Psychiatry, University College London, UK; 6 Universidad Cooperativa de Colombia, Medellín, Antioquia, Colombia; 7 Department of Psychological and Brain Sciences, Boston University, MA, USA; 8 South London and Maudsley NHS Foundation Trust, UK

**Keywords:** Conflict, war, mental health, psychiatry

## Abstract

**Background:**

The Colombian armed conflict has endured for almost 60 years. Colombia has a national psychosocial support service, called PAPSIVI, which is the largest initiative in history to address the psychosocial needs of civilians.

**Aims:**

Understand the extent to which PAPSIVI reaches priority groups and provides access to psychosocial support for victims of the conflict, something only previously tested in small studies.

**Methods:**

We used anonymised data from the register of victims of the armed conflict, including over 8 million individuals, with linked data to identify PAPSIVI access at the national level. We conducted univariable logistic regressions and multilevel logistic regression models adjusted for a range of potential confounders to examine associations.

**Results:**

Higher odds of PAPSIVI access were observed for females, those who register sex as ‘other’, older people, individuals from ethnic minorities, those on subsidised healthcare regimes and people with disabilities. Many specific exposures to the armed conflict were associated with increased levels of PAPSIVI access, including some of those reflecting the most severe exposures – namely, sexual violence, torture, physical injury, psychological injury and forced disappearance. Nevertheless, those affected by confinement, child recruitment to armed groups and homicide showed reliably lower levels of PAPSIVI access. In key marginalised groups, exposure to displacement and sexual violence was associated with lower rates of access.

**Conclusions:**

PAPSIVI successfully provides access to mental health support for key demographic groups and for a range of exposures to the armed conflict although areas of under-access indicate the need to better understand and address barriers to care.

## Introduction

Colombia has experienced a protracted and extensive armed conflict for nearly six decades. Despite a 2016 peace accord (Galán, 2021) between the government and the former armed group the Revolutionary Armed Forces of Colombia (in Spanish, *Fuerzas Armadas Revoluncionarias de Colombia – Ejercito del Pueblo*, or FARC-EP), the conflict is ongoing with several active armed groups still participating. Between 5 million (Internal Displacement Monitoring Centre, 2020) and 8 million (Unidad para la Atención y la Reparación Integral a las Víctimas, 2021) people are estimated to have been internally displaced by the conflict – many experiencing the lasting effects of violence, torture, massacres and widespread human rights abuses. Consequently, millions are thought to have mental health needs directly arising from the long-running and systemic effects of violence (Arenas et al., 2016; Idrobo et al., 2018; Ministerio de Salud, 2022; Tamayo Martínez et al., 2016).

Colombia has developed an ongoing programme to formalise the rights of and provide reparations to civilian victims of the conflict (García-Godos & Lid, 2010). This involves statutory registration as a recognised victim, following a process where individuals apply, have their claim evaluated and have their type of victimisation recorded, subsequently allowing access to reparations. As part of this, a country-wide programme, known by its Spanish-language acronym PAPSIVI (*Programa de Atención Psicosocial y Salud Integral a Víctimas*; in English, the Comprehensive Psychosocial Care and Health Programme for Victims), was launched in 2013 to provide support, including psychosocial support and psychological interventions for victims of the conflict. PAPSIVI provides individual psychological interventions, therapeutic work with families and group and community work, led by social workers, psychologists and community advocates (Ministerio de Salud y Protección Social, 2017a). The programme aims to treat mental health problems arising from the conflict but also address social and community problems where the conflict has affected community trust and cohesion (Ministerio de Salud y Protección Social, 2017b). By 2020, PAPSIVI had provided services to over 430,000 victims of the conflict, (Oficina Asesora de Planeación y Estudios Sectoriales & Oficina de Promoción Social, 2020) and this figure has grown to over 530,000 by 2023.

However, concerns have been raised about whether PAPSIVI is being accessed by the most severely affected and whether it is equally accessible to all victims of the conflict (Ramos-Vidal et al., 2022; Tamayo-Agudelo & Bell, 2019). This follows broader concerns about the extent to which socio-demographic (Abello-Llanos et al., 2009), geographic (Monsalve et al., 2022) and administrative issues (González-Uribe et al., 2022) are barriers to accessing support services for those affected by conflict.

One potentially important source of inequality relates to differential exposure to armed conflict. Although PAPSIVI aims to address the psychosocial needs of all victims of the conflict, certain types of exposure are potentially associated with greater needs alongside additional barriers to access. For example, more violent and individualised victimisation is associated with greater risk for subsequent mental health problems and disability (Trujillo et al., 2021) and is more common in more socially marginalised groups (Cuartas Ricaurte et al., 2019; León-Giraldo et al., 2021). Other types of exposures, like theft of resources or confinement, may cause practical barriers by preventing travel. Understanding how such barriers affect access is considered crucial to addressing inequalities, given that achieving first contact is considered an important milestone in successful engagement with services (Steel et al., 2006; Tansella & Micciolo, 1998).

In 2020, the Colombian Ministry of Health published an initial analysis of Phase I of the PAPSIVI psychosocial assistance programme, reporting high levels of acceptability following a survey and focus groups with 771 PAPSIVI attenders (Oficina Asesora de Planeación y Estudios Sectoriales & Oficina de Promoción Social, 2020). However, the complete service data has not been analysed, and the extent to which inequalities in accessing the PAPSIVI psychosocial support services are associated with sociodemographic characteristics and exposure to different aspects of the conflict is currently unknown.

Consequently, to better understand this, we used anonymised data from the register of victims of the armed conflict with linked data on PAPSIVI access to understand to what extent i) socio-demographic factors and ii) type of conflict exposure, are associated with access to PAPSIVI.

## Methods

The project received ethical approval from both the Ethics Committee of the Fundación Santa de Bogotá in Colombia (ID CCEI-15951-2023) and the University College London Research Ethics Committee in the United Kingdom (Project ID: 8275/002).

### Data sources

We used anonymised data from the central registry of victims of the Colombian armed conflict, known in Spanish as the *Registro Unico de Victimas* (RUV). This is a statutory list of victims of the armed conflict, maintained by the Colombian government, where registration as a victim of the conflict is a legal requirement for access to reparations. Registration involves attending approved points of registration that can include local government offices or offices of the Unit for Comprehensive Care and Reparation for Victims (in Spanish, *Unidad para la Atención y Reparación Integral a las Víctimas*). Individuals are interviewed and are asked for details of the time, location and context of the ‘victimising events’. Assessors may request additional documents to support their declaration. Victims are defined as having experienced victimisation themselves or as a first-degree family member (parents, children and siblings) of someone affected by a victimising event. The declaration is evaluated by the Victims Unit (in Spanish, la *Unidad para las Víctimas*) and the decision regarding successful registration as a victim is communicated to the applicant. Registered victim status is granted regardless of whether a criminal perpetrator is identified. Individuals may appeal within 5 days if their registration is refused. The registry of victims includes demographics and location of registration as well as type of victimisation. Variables used in this analysis are outlined below. This data was linked with data on access to PAPSIVI, indicating which individuals on the register had first contact with PAPSIVI psychosocial support services, defined as any single completed session. The data used by this study covers 2013 to 2021 and includes 8,359,538 registered individuals, of whom 534,819 individuals accessed PAPSIVI services for victims of the conflict.

### Measures

#### Outcome variables

We considered any access to PAPSIVI psychosocial support services, defined as attendance to any single PAPSIVI psychosocial support session, as our outcome variable. PAPSIVI services are only open to registered victims of the armed conflict and attendance is recorded through the presentation of the Colombian national ID card, allowing attendance to be linked to registered victim status.

#### Exposure variables

Registration in the victims’ registry (RUV) includes classification of exposure types as per the following officially recognised categories: witness to terrorism or combat, sexual violence, forced disappearance, forced displacement, homicide, exposure to mines/improvised explosives, kidnapping, torture, child recruitment to armed groups, forced land abandonment/dispossession, loss of personal belongings, physical injuries, psychological injuries, threats and confinement. Classification of exposure type is completed by assessors and follows criteria in the Manual of Evaluation Criteria (Unidad para las Víctimas, 2016). The original Spanish language categories and their translations are shown in Supplemental Table S1.

The majority of individuals are registered with a single victimisation type of conflict exposure and only one victimisation type is needed for official registration. However, some individuals have multiple types of victimisations registered, including different victimisations recorded at later dates or locations, and sometimes at multiple dates and locations. The reason for re-registration is not recorded and, therefore, it is not clear to what extent these are re-registrations are due to moving area, further victimisations or are erroneous. To address this, we used all recorded victimisation types as our primary exposure in our main analysis and we conducted a sensitivity analysis (described below) where only first recorded victimisation type was used as the exposure variable.

#### Covariates

We included socio-demographic variables (sex, age, ethnicity) as covariates in regression analyses to account for known demographic inequalities in exposure to the conflict and access to healthcare for people affected by the armed conflict in Colombia. Gender inequalities in exposure to the conflict and access to healthcare have been well documented in armed conflict generally (Bwirire et al., 2022) and Colombia specifically (Rivillas et al., 2018; Svallfors, 2024). Notably, sex was recorded as three categories in the original dataset: male, female and ‘LGBTI’. Given that LGBTI typically refers to ‘lesbian, gay, bisexual, trans and intersex’ and there was no separate variable to record sexuality, we have described this category as ‘other’ throughout and assume it refers to a minority sex or gender identity. Exposure to the conflict varies by age with younger individuals more likely to be exposed to violence and additional challenges in accessing healthcare experienced by the elderly (Cuartas Ricaurte et al., 2019; Gómez et al., 2009). Age was operationalised as a continuous variable. Ethnic minority communities have considerably higher rates of exposure to the conflict existing alongside long-standing inequalities in access to healthcare (Franco et al., 2006; Gómez-Restrepo et al., 2016).

There was no direct measure of socioeconomic status therefore we used healthcare regime as a proxy covariate as this is based on a poverty index which considers socioeconomic, demographic and welfare factors. People with lower levels of socioeconomic resources have higher levels of exposure to the conflict and lower access to healthcare (Cuartas Ricaurte et al., 2019). Healthcare in Colombia is a mixed public-private model with a subsidised health insurance option for those eligible based on the poverty index. Healthcare regime was recorded as subsidised or contributory in the dataset.

We also included a measure of mean conflict intensity for the 10 years before the start of the PAPSIVI programme at the municipality of registration of each individual, based on Centre for Resource for Analysis of Armed Conflict (CERAC) database (Restrepo et al., 2003). The conflict has impacted healthcare provision in the most affected areas by preventing state healthcare provision, damaging healthcare facilities, and dissuading staff from working in the most affected areas, leading to losing confidence in services (Bernal et al., 2024). CERAC’s conflict intensity measure is based on the presence of armed actors by year and the number of conflict events at the municipality level in Colombia and categorised into high or low intensity compared to the national average

#### Other variables

Disability at the time of registration was not included as a covariate as it may be on the causal pathway between conflict exposure and PAPSIVI access, but its unadjusted association with PAPSIVI access is reported. Disability is reported as present or absent and is based on self-declaration at time of registration with the option for assessors to request medical documentation or a statutory ‘certificate of disability’ to verify status.

Location of registration was recorded by municipality, which are 1104 administrative sub-regions of Colombia with boundaries broadly based on population density.

### Statistical analysis

Analysis was completed using *R* version 4.4.1 (R Core Team, 2020) on a Windows x86_64 secure analysis platform. All analysis code is available on the online archive: https://github.com/vaughanbell/papsivi_access.

Descriptive statistics were generated as frequencies and means with standard deviations, and we also plotted the geographical distribution of a total number of registered victims and PAPSIVI attendees as a proportion of victims using the *colmaps* package (Clavijo et al., 2015). Univariable logistic regression was used to examine associations between sex, ethnicity, disability, healthcare regime and access to any access to PAPSIVI. Multilevel logistic regression using the *glmmTMB* package (Brooks et al., 2017) was used to estimate associations between type of victimisation and access to PAPSIVI. We first ran each model unadjusted (model 1), then adjusted for sex, age and healthcare regime, ethnicity (model 2). Finally, we also included municipality CERAC exposure to conflict (model 3). As exposure to the armed conflict is not distributed evenly and individuals nested within the same geographical areas are likely to have correlated observations, municipality was included as a random-effects (clustering) factor to account for geographical similarities in exposure. For analysis including multiple registration, participant was also included as a random-effects factor in the multi-level models to account for similarity within individuals. We additionally tested for interaction between exposure type, and sex, healthcare regime and victimisation.

#### Sensitivity analysis

Given the reasons for multiple registrations are not recorded in the data, we investigated whether results differed when defining the conflict exposure using the first recorded victimisation type only as the main exposure.

## Results

### Participants

All 8,359,538 registered individuals and 534,819 individuals who accessed PAPSIVI were included in analysis.

### Descriptive statistics

Demographic and descriptive statistics are shown in Table 1.

**Table 1.** Descriptive statistics.

Characteristic	All RUV registered victims		Accessed PAPSIVI	
	Total *N* = 8,359,538		Total *N* = 534,818	
	*N*	%	N	%
Sex				
Male	4,240,400	50.70	201,666	37.70
Female	4,114,735	49.20	332,811	62.20
Other	3,856	<0.01	321	0.10
Missing	547	<0.01	20	<0.01
Ethnicity				
Indigenous	1,061,174	12.70	96,282	18.00
Roma	223,058	2.70	17,776	3.30
Raizal (San Andres and Providencia)	7,280	0.10	469	0.10
Negro, Mulato, Afrocolombiano descenciente	10,426	0.10	827	0.20
Palenquero de San Basilio	8,885	0.10	415	0.10
White/Mestiza	7,048,715	84.30	419,049	78.40
Healthcare regime				
Contributory	1,987,877	23.80	100,394	18.80
Subsidised	5,088,138	60.90	402,431	75.20
Missing	1,283,523	15.40	31,993	6.00
Disability				
No	7,967,800	95.30	495,374	92.60
Yes	391,738	4.70	39,444	7.40
Age				
	*M*	*SD*	*M*	*SD*
	34.7	20.6	41.2	19.1
Missing	25,217	0.30%	140	<0.01%

*Note*. PAPSIVI = Comprehensive Psychosocial Care and Health Programme for Victims (Programa de Atención Psicosocial y Salud Integral a Víctimas); RUV = Central Registry for Victims (Registro Unico de Victimas).

Out of 8,359,538 individuals who registered as victims with the RUV up to the year 2021, 534,818 (6.4%) accessed PAPSIVI services. Most registered victims were on a subsidised healthcare regime (60.9%) and not registered disabled (95.3%). The mean age of registered victims was 34.7 years old, while the mean age of registered victims who accessed PAPSIVI was 41.2 years old.

**Figure 1.**
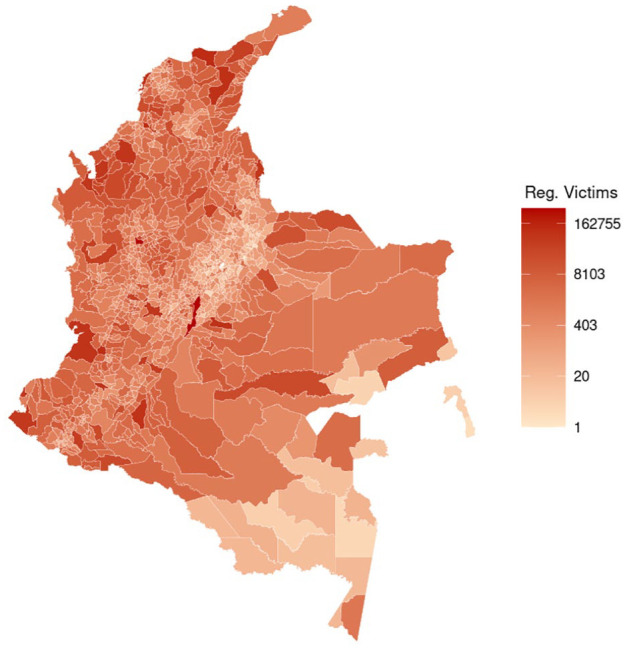
Registered victims of the armed conflict in Colombia by municipality of registration (log scale).

**Figure 2.**
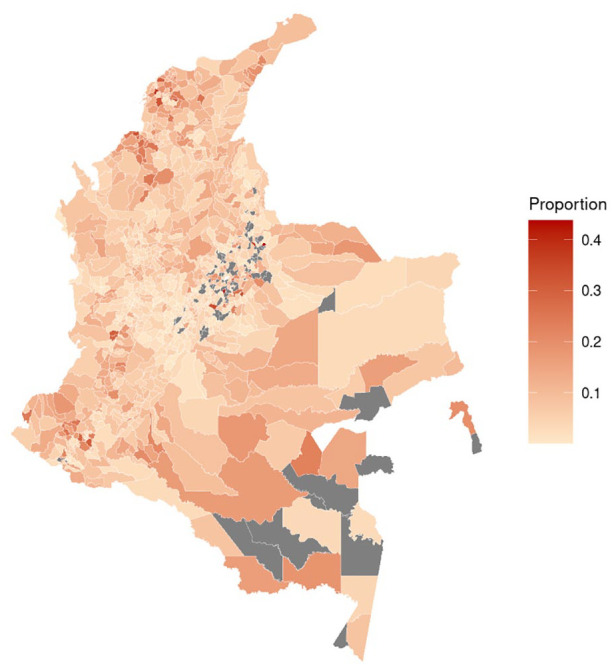
Proportion of registered victims of the conflict accessing PAPSIVI by municipality of registration 2013 to 2021. Grey = no municipality data.

Figure 1 shows the log scale distribution of registered victims of the armed conflict in Colombia by municipality and Figure 2 the proportion of registered victims who accessed PAPSIVI in each municipality. Supplemental Figure S1 shows distribution of PAPSIVI access by municipality.

### Sociodemographic characteristics and PAPSIVI access

As shown in Table 2, the odds of accessing PAPSIVI treatment increased with age. Furthermore, females, those with sex classified as ‘Other’, individuals with a disability, those under a subsidised healthcare regime, and people from ethnic minority had higher odds of accessing PAPSIVI.

**Table 2.** Unadjusted associations between sex, healthcare regime, disability, ethnicity, mental health diagnosis and age and PAPSIVI access.

Characteristic	OR [95% CI]
Sex	
Female	1.76 [1.75, 1.77]
Other	1.82 [1.62, 2.04]
Age	1.016 [1.015, 1.016]
Ethnicity	
Minority	1.53 [1.52, 1.54]
Healthcare regime	
Subsidised	1.61[1.60, 1.63]
Disability	
Yes	1.69 [1.67, 1.71]

OR = odds ratio.

### Association between conflict exposure type and PAPSIVI access

**Figure 3.**
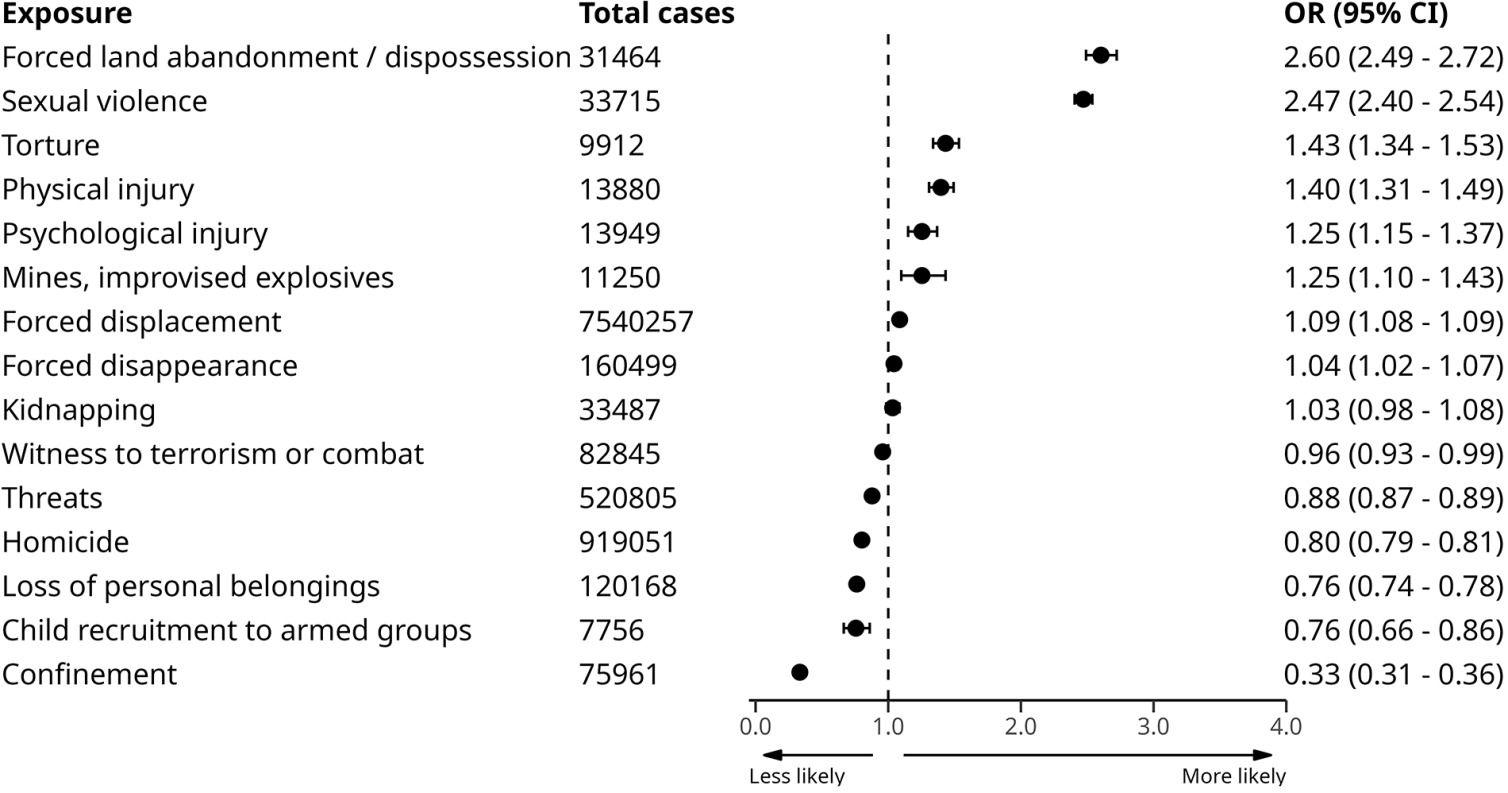
Association between conflict exposure types and PAPSIVI access from model 3 (adjusted for age, sex, healthcare regime and CERAC conflict intensity at location of registration). *Note*. OR = odds ratio; 95% CI = 95% confidence intervals.

Results for the adjusted, multi-level model associations between conflict exposure type and PAPSIVI access, ordered by strength of association, are displayed in Figure 3. The full results of all models are reported in Supplemental Table S2.

We additionally tested for interactions in our adjusted model 3 between conflict exposure type and (i) sex, (ii) healthcare regime, and (iii) ethnic minority status, and we highlight results which contradict the general association between each of these factors and increased PAPSIVI access. Full stratified results are reported Supplemental Tables S4, S5 and S6.

For forced land abandonment and dispossession, there were lower odds of PAPSIVI access for females (OR = 2.41; 95% CI [2.26, 2.57]) and people with sex registered as ‘Other’ (OR = 2.00; 95% CI [0.19, 20.93]), compared to males (OR = 2.83; 95% CI [2.67, 3.01]). For displacement, there were slightly low odds of PASIVI access for females (OR = 1.06; 95% CI [1.05, 1.07]) and markedly lower odds of PAPSIVI for people with sex registered as ‘Other’ (OR = 0.72; 95% CI [0.60, 0.86]) compared to males (OR = 1.17; 95% CI [1.15, 1.18]). For sexual violence, although there were higher odds of PAPSIVI access for females (OR = 2.49; 95% CI [2.42, 2.56]) compared to males (OR = 2.28; 95% CI [1.96, 2.65]), those with sex registered as ‘Other’ showed considerably lower odds of access (OR = 1.43; 95% CI [1.10, 1.86]).

When compared to people with contributory healthcare, those on a subsidised healthcare regime were less likely to access PAPSIVI for threats and confinement but the absolute difference was small (less than 0.02 odds difference). However, there was substantially lower odds of PAPSIVI access for displacement (subsidised OR = 1.05; 95% CI [1.04, 1.06]/contributory OR = 1.20; 95% CI [1.18, 1.22]), mines and improvised explosives (subsidised OR = 1.15; 95% CI [0.99, 1.34]/contributory OR = 1.65; 95% CI [1.28, 2.14]), child recruitment to armed groups (subsidised OR = 0.69; 95% CI [0.59, 0.80]/(contributory OR = 0.96; 95% CI [0.76, 1.22]) and sexual violence (subsidised OR = 2.27; 95% CI [2.20, 2.35]/contributory OR = 3.13; 95% CI [2.97, 3.30]).

Those from ethnic minorities were less likely to access PAPSIVI after exposure to confinement (minority ethnicity OR = 0.25; 95% CI [0.23, 0.28]/majority ethnicity OR = 0.50; 95% CI [0.45, 0.55]), displacement (minority ethnicity OR = 0.93; 95% CI [0.92, 0.95]/majority ethnicity OR = 1.12; 95% CI [1.11, 1.13]), forced land abandonment and dispossession (minority ethnicity OR = 2.37; 95% CI [2.05, 2.74]/majority ethnicity OR = 2.59; 95% CI [2.48, 2.72]) and sexual violence (minority ethnicity OR = 2.30; 95% CI [2.18, 2.42]/majority ethnicity OR = 2.53; 95% CI [2.45, 2.61]).

### Sensitivity analysis

**Figure 4.**
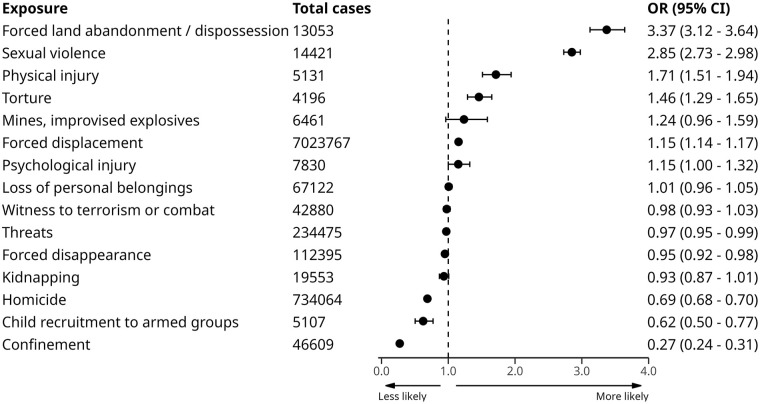
Association between first recorded conflict exposure type and PAPSIVI access from Model 3 (adjusted for age, sex, healthcare regime and CERAC conflict intensity at location of registration). *Note*. OR = odds ratio; 95% CI = 95% confidence intervals.

Results for the sensitivity analysis when including only the first recorded conflict exposure is displayed in Figure 4. The full results of all models are reported in Supplemental Table S3. As can be seen from Figure 4, the strengths of associations are slightly increased with wider confidence intervals but the overall pattern of results is very similar.

## Discussion

In over 8 million registered civilian victims of the armed conflict in Colombia, we report higher level of access to the national PAPSIVI psychosocial support programme for females, minority sex and gender individuals, older people, individuals from ethnic minorities, those on subsidised healthcare regimes and people with disabilities. We also report that many exposures to the armed conflict are associated with increased levels of PAPSIVI access, present in both the main and sensitivity analyses, including some of those likely reflecting the most severe exposures, namely, sexual violence, torture, physical injury, psychological injury and forced disappearance. Nevertheless, those affected by confinement, child recruitment to armed groups and homicide showed reliably lower levels of PAPSIVI access, indicating the need to better understand and address barriers to pathways to care in affected individuals.

Two exposures to the armed conflict were associated with the highest odds of PAPSIVI access, namely ‘forced land abandonment and dispossession’ and ‘sexual violence’. The latter, conflict-related sexual violence, has particularly severe consequences on individual victims and communities, as evidenced both by international studies (Ba & Bhopal, 2017) and those from Colombia where high rates of psychological distress (Torres et al., 2020; Zamora-Moncayo et al., 2021) and, particularly, posttraumatic distress disorder (Barchelot Aceros et al., 2023) have been reported. Land dispossession has not traditionally been identified as a priority cause of poor mental health but increasing evidence has identified wide-ranging and profound effects (Ninomiya et al., 2023) due to the parallel effects of loss to the individual and loss of community networks, culture and rootedness. In Colombia, conflict-related land dispossession can be present with other exposures including sexual violence and torture (Arias-Vanegas & Caicedo-Fernández, 2017; Céspedes-Báez, 2010) and has a range of enduring socioeconomic effects on affected individuals, including loss of livelihood and housing, disrupted education and breakdown of social networks (Chávez Plazas & Romero Picón, 2010), acting as a risk factor for poor mental health and psychiatric disorder (Polanco & Barrero, 2018; Shultz et al., 2014). These ‘additive’ effects of land dispossession, leading to a range of complex needs, may explain why those who register as being victimised by land dispossession access PAPSIVI at higher odds than those who register as being victimised by ‘loss of belongings’ and ‘displacement’.

The fact that exposures to some of the most severe effects of the conflict are associated with higher odds of access to PAPSIVI is promising, although it is worth considering why some exposures are associated with lower odds of access. Confinement, child recruitment to armed groups and homicide were reliably associated with lower odds of PAPSIVI access in both the main and sensitivity analysis. The reasons for these remain unclear and need to be the subject of further investigation, however, we suggest some hypotheses here. People who have been exposed to child recruitment either personally, or through having had a first-degree child or adolescent family member recruited by armed groups, may be concerned about consequences of seeming to engage with state services. Confinement refers to situations where typically whole communities have been confined by armed groups who can control significant areas of territory, and it may be that attending subsequent PAPSIVI sessions after registering as a victim is additionally difficult because of restrictions on travel in general, or restrictions based on reasons for travel.

The reasons for reduced access for people who have homicide exposure is less clear. Homicide is a second-person victimisation for PAPSIVI attendees and although fear about consequences may be a factor, this should also be the case for other exposures (such as torture and sexual violence) that have far higher odds of access. Nevertheless, qualitative work on victims of the armed conflict in Colombia has reported a range of impacts and consequences including psychological distress, unemployment and economic difficulties, stigma and breakdown of social ties (Burgess & Fonseca, 2020; Zamora-Moncayo et al., 2021). These impacts likely interact and probably depend on complex relationships between exposure, community, resources and pathways to care that may mean straightforward explanations may not be apparent for all exposures, and this requires further investigation.

PAPSIVI appears successful in providing higher levels of access to women and girls, minority sex and gender individuals, older people, individuals from ethnic minorities, people on subsidised healthcare regimes and those with disabilities, who all, on average, access PAPSIVI at higher levels. These groups have been identified as having greater mental health needs in relationship to the conflict, due to the effects of the conflict being compounded by pre-existing social marginalisation, leading to higher reported levels of psychiatric disorder and psychopathology (León-Giraldo et al., 2023; Tamayo Martínez et al., 2016). Consequently, they have been identified as being priority populations for psychosocial support (Castillejo Cuéllar et al., 2022a; Cuartas Ricaurte et al., 2019; Garavito et al., 2023).

Nevertheless, interaction analyses identified some armed conflict exposures that were identified with lower PAPSIVI access within these on-average higher access groups. Perhaps most notably, people who identified as ‘other’ for their sex, those on a subsidised healthcare regime and people from ethnic minorities were all less likely to access PAPSIVI following exposure to sexual violence. The extent to which conflict-related sexual violence have differentially affected demographic groups in Colombia beyond gender is not clear due to a lack of studies with representative sampling, and, indeed, the difficulty of conducting such studies. Nevertheless, queer, low income and ethnic minority individuals have been widely identified as bearing a disproportional impact of sexual violence from the armed conflict (Castillejo Cuéllar et al., 2022b; Oxfam, 2009). In addition, they report exclusion from support services, something that has been described as a form of ‘secondary victimisation’ (Gualdron & Steward, 2015). Qualitative studies have described a range of impacts, barriers to service access and resilience factors in marginalised groups affected by the conflict that are often particular to their context and experience (Tamayo Acevedo et al., 2020; Zamora-Moncayo et al., 2021) and it is likely that initiatives to address these barriers will need to be specific to the affected communities to address their specific needs.

It is important to highlight several limitations to this study. Firstly, the data used in this study solely represent people who have registered with the Colombian state as victims of the armed conflict. Those who have not registered are additionally excluded from statutory support services and may have different profiles of conflict exposure, needs and barriers to support. The analysis consequently relies on variables collected during standard service operation and is limited to existing data and previous data collection method, meaning analyses could be affected by residual confounding. As noted, the original coding of the ‘Other’ category for the recoding of sex was ‘LGBTI’. We have interpreted this as ‘minority sex and gender’ but the extent to which this was used by registrants or assessors to classify a range of queer identities remains unclear. Similarly, although healthcare regime is assessed on calculation of a poverty index, the only variable available was healthcare regime as a binary outcome, potentially reduced the ability to more finely control for a wider range of socioeconomic status. While we had the municipality of registration as a victim of the conflict, we did not have data on location of the PAPSIVI centre that each individual attended. PAPSIVI has been designed to provided attention across the country and near to points of registration, meaning the majority of attendees are likely to have attended ‘locally’. However, not being able to control for distance from registration to PAPSIVI consultation means we were limited in being able to examine potential confounders related to travel and accessibility.

The results from this study suggest that PAPSIVI is largely achieving its aims in terms of providing access to psychosocial support services for victims of the armed conflict in Colombia. However, understanding the quality of service received by attendees is also important to fully evaluate its effectiveness. Additional analysis could investigate to what extent the different types of intervention are being appropriately offered to victims of the conflict based on their profile of exposure, and whether patients are receiving adequate courses of treatment or dropout of treatment early. In addition, PAPSIVI does not routinely collect outcome data in the form of mental health measures that would help evaluate the extent to which the treatment is effective. This has previously been highlighted as a priority (Tamayo-Agudelo & Bell, 2019) and we further highlight its use here to support future evaluation and service development.

## Conclusions

In conclusion, we report associations between demographic characteristics and exposures to the armed conflict for access to the PAPSIVI psychosocial support service in over 8 million victims of the armed conflict. PAPSIVI showed significant success in providing higher levels of access to groups highlighted as having greater conflict-related needs. Nevertheless, victims who were exposed to confinement, child recruitment to armed groups and homicide appear to be under-accessing PAPSIVI, and findings relating to reduced access following exposure to sexual violence and displacement across several traditionally marginalised populations should raise questions about the need for additional outreach and the extent to which services are well-adapted to these groups.

## Supplemental Material

sj-docx-1-isp-10.1177_00207640251336726 – Supplemental material for Armed conflict exposure types are not equally associated with access to psychosocial support: A study of over 8 million victims of the Colombian armed conflict Supplemental material, sj-docx-1-isp-10.1177_00207640251336726 for Armed conflict exposure types are not equally associated with access to psychosocial support: A study of over 8 million victims of the Colombian armed conflict by Charlotte Constable Fernandez, Alida Acosta-Ortiz, María Camila García Durán, Rob Saunders, Francesca Solmi, William Tamayo-Agudelo, Fabio Idrobo and Vaughan Bell in International Journal of Social Psychiatry

## References

[bibr1-00207640251336726] Abello-Llanos R. Macías M. A. Blanco-Abarca A. Madariaga-Orozco C. Manrique-Palacio K. Martínez-González M. Turizo-Palencia Y. Díaz-Méndez D. (2009). Bienestar y trauma en personas adultas desplazadas por la violencia política. Universitas Psychologica, 8(2), 455–470.

[bibr2-00207640251336726] Arenas A. Gómez-Restrepo C. Rondón M. (2016). Factores asociados a la conducta suicida en Colombia. Resultados de la Encuesta Nacional de Salud Mental 2015. Revista Colombiana de Psiquiatría, 45, 68–75. 10.1016/j.rcp.2016.03.006 27993258

[bibr3-00207640251336726] Arias-Vanegas J. Caicedo-Fernández A. (2017). Etnografías e historias de despojo: Una introducción. Revista Colombiana de Antropología, 53(1), 7–22.

[bibr4-00207640251336726] Ba I. Bhopal R. S. (2017). Physical, mental and social consequences in civilians who have experienced war-related sexual violence: A systematic review (1981–2014). Public Health, 142, 121–135. 10.1016/j.puhe.2016.07.019 27622295

[bibr5-00207640251336726] Barchelot Aceros L. J. Pabón Poches D. K. Mieles Toloza I. L. Galván Patrignani G. D. Barchelot Aceros L. J. Pabón Poches D. K. Mieles Toloza I. L. Galván Patrignani G. D . (2023). TEPT en sobrevivientes de violencia o abuso sexual en el conflicto armado colombiano. Psicoperspectivas, 22(3), 53–69. 10.5027/psicoperspectivas-vol22-issue3-fulltext-2967

[bibr6-00207640251336726] Bernal O. Garcia-Betancourt T. León-Giraldo S. Rodríguez L. M. González-Uribe C. (2024). Impact of the armed conflict in Colombia: Consequences in the health system, response and challenges. Conflict and Health, 18(1), Article 4. 10.1186/s13031-023-00561-6 PMC10762784 38172982

[bibr7-00207640251336726] Brooks M. E. Kristensen K. Van Benthem K. J. Magnusson A. Berg C. W. Nielsen A. Skaug H. J. Machler M. Bolker B. M. (2017). GlmmTMB balances speed and flexibility among packages for zero-inflated generalized linear mixed modeling. The R Journal, 9(2), 378–400.

[bibr8-00207640251336726] Burgess R. A. Fonseca L. (2020). Re-thinking recovery in post-conflict settings: Supporting the mental well-being of communities in Colombia. Global Public Health, 15(2), 200–219. 10.1080/17441692.2019.1663547 31526162

[bibr9-00207640251336726] Bwirire D. Crutzen R. Namegabe E. N. Letschert R. Vries N. de . (2022). Health inequalities in post-conflict settings: A systematic review. PLOS ONE, 17(3), Article e0265038. 10.1371/journal.pone.0265038 PMC8920275 35286351

[bibr10-00207640251336726] Castillejo Cuéllar A. Franco Agudelo S. Ganem Maloof K. de Roux Rengifo F. J . (2022a). Hallazgos y recomendaciones de la Comisión de la Verdad de Colombia. Comisión de la Verdad de Colombia.

[bibr11-00207640251336726] Castillejo Cuéllar A. Franco Agudelo S. Ganem Maloof K. de Roux Rengifo F. J . (2022b). Mi cuerpo es la verdad: Experiencias de mujeres y personas LGBTIQ+ en el conflicto armado. Comisión de la Verdad de Colombia.

[bibr12-00207640251336726] Céspedes-Báez L.-M. (2010). La violencia sexual en contra de las mujeres como estrategia de despojo de tierras en el conflicto armado colombiano. Estudios Socio-Jurídicos, 12(2), 273–304.

[bibr13-00207640251336726] Chávez Plazas Y. Romero Picón Y . (2010). Entre el despojo y el destierro: Una aproximación al problema de la tierra en familias desplazadas por la violencia en colombia. Tabula Rasa, 12, 159–173.

[bibr14-00207640251336726] Clavijo A. Cruz J. Moreno D. (2015). Colmaps [R]. nebulae-co. https://github.com/nebulae-co/colmaps

[bibr15-00207640251336726] Cuartas Ricaurte J. Karim L. L. Martínez Botero M. A. Hessel P . (2019a). The invisible wounds of five decades of armed conflict: Inequalities in mental health and their determinants in Colombia. International Journal of Public Health, 64(5), 703–711. 10.1007/s00038-019-01248-7 31119303

[bibr16-00207640251336726] Franco S. Suarez C. M. Naranjo C. B. Báez L. C. Rozo P. (2006). The effects of the armed conflict on the life and health in Colombia. Ciência & Saúde Coletiva, 11(2), 349–361. 10.1590/S1413-81232006000200013

[bibr17-00207640251336726] Galán E. C. D. (2021). El Acuerdo de Paz para Colombia. Un singular mecanismo de consolidación de la paz. Anuario Mexicano de Derecho Internacional, 21, 933–961. 10.22201/iij.24487872e.2021.21.15614

[bibr18-00207640251336726] Garavito G. A. A. Burgess R. Sanguinetti M. C. D. Peters L. E. R. Juan N. V. S. (2023). Mental health services implementation in Colombia–A systematic review. PLOS Global Public Health, 3(12), Article e0001565. 10.1371/journal.pgph.0001565 PMC10699609 38055705

[bibr19-00207640251336726] García-Godos J. Lid K. A. O. (2010). Transitional justice and victims’ rights before the end of a conflict: The unusual case of Colombia. Journal of Latin American Studies, 42(3), 487–516. 10.1017/S0022216X10000891

[bibr20-00207640251336726] Gómez F. Curcio C.-L. Duque G. (2009). Health care for older persons in Colombia: A country profile. Journal of the American Geriatrics Society, 57(9), 1692–1696. 10.1111/j.1532-5415.2009.02341.x 19515103

[bibr21-00207640251336726] Gómez-Restrepo C. Rincón C. J. Urrego-Mendoza Z. (2016). Salud mental, sufrimiento emocional, problemas y trastornos mentales de indígenas colombianos. Datos de la Encuesta Nacional de Salud Mental 2015. Revista Colombiana de Psiquiatría, 45, 119–126. 10.1016/j.rcp.2016.09.005 27993246

[bibr22-00207640251336726] González-Uribe C. Olmos-Pinzón A. León-Giraldo S. Bernal O. Moreno-Serra R. (2022). Health perceptions among victims in post-accord Colombia: Focus groups in a province affected by the armed conflict. PLoS ONE, 17(3), Article e0264684. 10.1371/journal.pone.0264684 PMC8890648 35235591

[bibr23-00207640251336726] Gualdron O. Steward F. (2015). Victimización y violencia sexual en el conflicto armado en Colombia. Subjetividad y Procesos Cognitivos, 19(2), 173–186.

[bibr24-00207640251336726] Idrobo F. Hessel P. Harker A. Evans-Lacko S. Avendaño M. (2018). Mental health of victims and ex-FARC members: A challenge for the peace process in Colombia. The Lancet Psychiatry, 5(6), 467–468. 10.1016/S2215-0366(18)30134-2 29857841

[bibr25-00207640251336726] Internal Displacement Monitoring Centre. (2020). Global report on internal displacement 2020. IDMC. https://www.internal-displacement.org/global-report/grid2020/

[bibr26-00207640251336726] León-Giraldo S. Casas G. Cuervo-Sánchez J. S. García T. González-Uribe C. Moreno-Serra R. Bernal O. León-Giraldo S. Casas G. Cuervo-Sánchez J. S. García T. González-Uribe C. Moreno-Serra R. Bernal O. (2023). Trastornos de salud mental en población desplazada por el conflicto en Colombia: Análisis comparado frente a la Encuesta Nacional de Salud Mental 2015. Revista Colombiana de Psiquiatría, 52(2), 121–129. 10.1016/j.rcp.2021.04.012 37453820

[bibr27-00207640251336726] León-Giraldo S. Casas G. Cuervo-Sanchez J. S. González-Uribe C. Bernal O. Moreno-Serra R. Suhrcke M. (2021). Health in conflict zones: Analyzing inequalities in mental health in colombian conflict-affected territories. International Journal of Public Health, 66, Article 595311. 10.3389/ijph.2021.595311 PMC8565266 34744562

[bibr28-00207640251336726] Ministerio de Salud. (2022). Salud mental: Asunto de todos. https://www.minsalud.gov.co/Paginas/Salud-mental-asunto-de-todos.aspx

[bibr29-00207640251336726] Ministerio de Salud y Protección Social. (2017a). Programa de Atención Psicosocial y Salud Integral a Víctimas del Conflicto Armado (PAPSIVI) Documento Marco. Oficina de Promoción Social.

[bibr30-00207640251336726] Ministerio de Salud y Protección Social. (2017b). Protocolo de Atención Integral en Salud con Enfoque Psicosocial a Víctimas de Conflicto Armado. Oficina de Promoción Social.

[bibr31-00207640251336726] Monsalve S. D. Vargas-Monroy A. M. Ariza J. E. Oñate Cuello A. M. Ropero Vera A. R. Bermudez Cuello J. C. Arzuaga Zuleta L. Cubillos Novella A. F. Peñaloza Quintero E. Fernández Ortiz Y. N. Carrillo M. A. Kroeger A. (2022). Mental health among displaced and non-displaced populations in Valledupar, Colombia: Do inequalities continue? Pathogens and Global Health, 116(5), 305–318. 10.1080/20477724.2021.1989186 34689701 PMC9248948

[bibr32-00207640251336726] Ninomiya M. E. M. Burns N. Pollock N. J. Green N. T. G. Martin J. Linton J. Rand J. R. Brubacher L. J. Keeling A. Latta A. (2023). Indigenous communities and the mental health impacts of land dispossession related to industrial resource development: A systematic review. The Lancet Planetary Health, 7(6), e501–e517. 10.1016/S2542-5196(23)00079-7 37286247

[bibr33-00207640251336726] Oficina Asesora de Planeación y Estudios Sectoriales, Oficina de Promoción Social. (2020). Evaluación Programa de Atención Psicosocial y Salud Integral a Víctimas–PAPSIVI Informe Final. Bogotá.

[bibr34-00207640251336726] Oxfam. (2009). Sexual Violence in Colombia. https://www.oxfam.org/en/research/sexual-violence-colombia

[bibr35-00207640251336726] Polanco J. G. C. Barrero J. A. C . (2018). Salud mental de los colombianos tras el posconflicto. In Polanco J. G. Castañeda Barrero J. A. Camargo (Eds.), Conflicto armado y salud mental: Una mirada al conflicto colombiano (pp. 21–31). Corporación Universitaria Minuto de Dios.

[bibr36-00207640251336726] R Core Team. (2020). R: A language and environment for statistical computing. R Foundation for Statistical Computing. https://www.R-project.org/

[bibr37-00207640251336726] Ramos-Vidal I. Palacio J. Villamil I. Uribe A. Domínguez E. Wehdeking I . (2022). Barreras en la implementación de programas centrados en comunidad desplazada: Tensiones entre teoría y praxis en el modelo PAPSIVI. In Arcón V. A. Bustos Molinares D. P. Mayor (Eds.), Desplazamiento: Perspectivas y Estrategias de Intervención Desde El Caribe Colombiano (pp. 101–140). Editorial Universidad del Norte.

[bibr38-00207640251336726] Restrepo J. A. Spagat M. Vargas J. F. (2003). The dynamics of the Colombian civil conflict: A new data set. SSRN 480247.

[bibr39-00207640251336726] Rivillas J. C. Rodriguez R. D. Song G. Martel A. (2018). How do we reach the girls and women who are the hardest to reach? Inequitable opportunities in reproductive and maternal health care services in armed conflict and forced displacement settings in Colombia. PLOS ONE, 13(1), Article e0188654. 10.1371/journal.pone.0188654 PMC5773007 29346375

[bibr40-00207640251336726] Shultz J. M. Ceballos Á. M. G. Espinel Z. Oliveros S. R. Fonseca M. F. Florez L. J. H. (2014). Internal displacement in Colombia: Fifteen distinguishing features. Disaster Health, 2(1), 13–24. 10.4161/dish.27885 28228997 PMC5314912

[bibr41-00207640251336726] Steel Z. Mcdonald R. Silove D. Bauman A. Sandford P. Herron J. Harry Minas I. (2006). Pathways to the first contact with specialist mental health care. Australian & New Zealand Journal of Psychiatry, 40(4), 347–354. 10.1080/j.1440-1614.2006.01801.x 16620317

[bibr42-00207640251336726] Svallfors S. (2024). Gender dynamics during the Colombian armed conflict. Social Politics: International Studies in Gender, State & Society, 31(2), 298–320. 10.1093/sp/jxad016

[bibr43-00207640251336726] Tamayo Acevedo M. I. Tamayo Acevedo L. S. Tamayo Acevedo L. H . (2020). La violencia se vive de miles maneras: Voces de mujeres víctimas de violencia sexual en el conflicto armado del Carmen de Bolívar - Región Caribe, Colombia, 2018–2019. Estudios sobre las culturas contemporáneas, 51, 9–34.

[bibr44-00207640251336726] Tamayo Martínez N. Rincón Rodríguez C. J. de Santacruz C. Bautista Bautista N. Collazos J. Gómez–Restrepo C . (2016). Problemas mentales, trastornos del afecto y de ansiedad en la población desplazada por la violencia en Colombia, resultados de la Encuesta Nacional de Salud Mental 2015. Revista Colombiana de Psiquiatría, 45, 113–118. 10.1016/j.rcp.2016.09.004 27993245

[bibr45-00207640251336726] Tamayo-Agudelo W. Bell V. (2019). Armed conflict and mental health in Colombia. BJPsych International, 16(2), 40–42. 10.1192/bji.2018.4 31144687 PMC6520540

[bibr46-00207640251336726] Tansella M. Micciolo R. (1998). Unplanned first contact as a predictor of future intensive use of mental health services. Social Psychiatry and Psychiatric Epidemiology, 33(4), 174–180. 10.1007/s001270050040 9567667

[bibr47-00207640251336726] Torres L. C. Maciel C. G. G. Mendoza A. L. G. Torres L. S. Acosta L. B. (2020). Malestar psicológico en víctimas de violencia sexual, intrafamiliar y del conflicto armado. Tempus Psicológico, 3(1), Article 1. 10.30554/tempuspsi.3.1.2878.2020

[bibr48-00207640251336726] Trujillo S. Giraldo L. S. López J. D. Acosta A. Trujillo N. (2021). Mental health outcomes in communities exposed to Armed Conflict Experiences. BMC Psychology, 9(1), Article 127. 10.1186/s40359-021-00626-2 PMC8394205 34452647

[bibr49-00207640251336726] Unidad para la Atención y la Reparación Integral a las Víctimas. (2021). Registro Único de Víctimas (RUV). Unidad para las Víctimas. https://www.unidadvictimas.gov.co/es/registro-unico-de-victimas-ruv/37394

[bibr50-00207640251336726] Unidad para las Víctimas. (2016). Manual Criterios de Valoración (Versión 2). https://www.unidadvictimas.gov.co/sites/default/files/documentosbiblioteca/manual-de-valoracion-v2.pdf

[bibr51-00207640251336726] Zamora-Moncayo E. Burgess R. A. Fonseca L. González-Gort M. Kakuma R. (2021). Gender, mental health and resilience in armed conflict: Listening to life stories of internally displaced women in Colombia. BMJ Global Health, 6(10), Articlr e005770. 10.1136/bmjgh-2021-005770 PMC8499256 34620613

