# Supplementary material for: Armed conflict exposure types are not equally associated with access to psychosocial support: A study of over 8 million victims of the Colombian armed conflict
Source: Int J Soc Psychiatry. 2025 May 27;71(7):1281–91. doi: 10.1177/00207640251336726 (PMC12547036; doi:10.1177/00207640251336726)
Supplement: sj-docx-1-isp-10.1177_00207640251336726 – Supplemental material for Armed conflict exposure types are not equally associated with access to psychosocial support: A study of over 8 million victims of the Colombian armed conflict [file sj-docx-1-isp-10.1177_00207640251336726.docx]

**Supplementary Material for**

**Armed conflict exposures are not equally associated with access to psychosocial support: a study of over 8 million victims of the Colombian armed conflict**

**Figure S1.** Distribution of PAPSIVI access by municipality of registration (log scale)


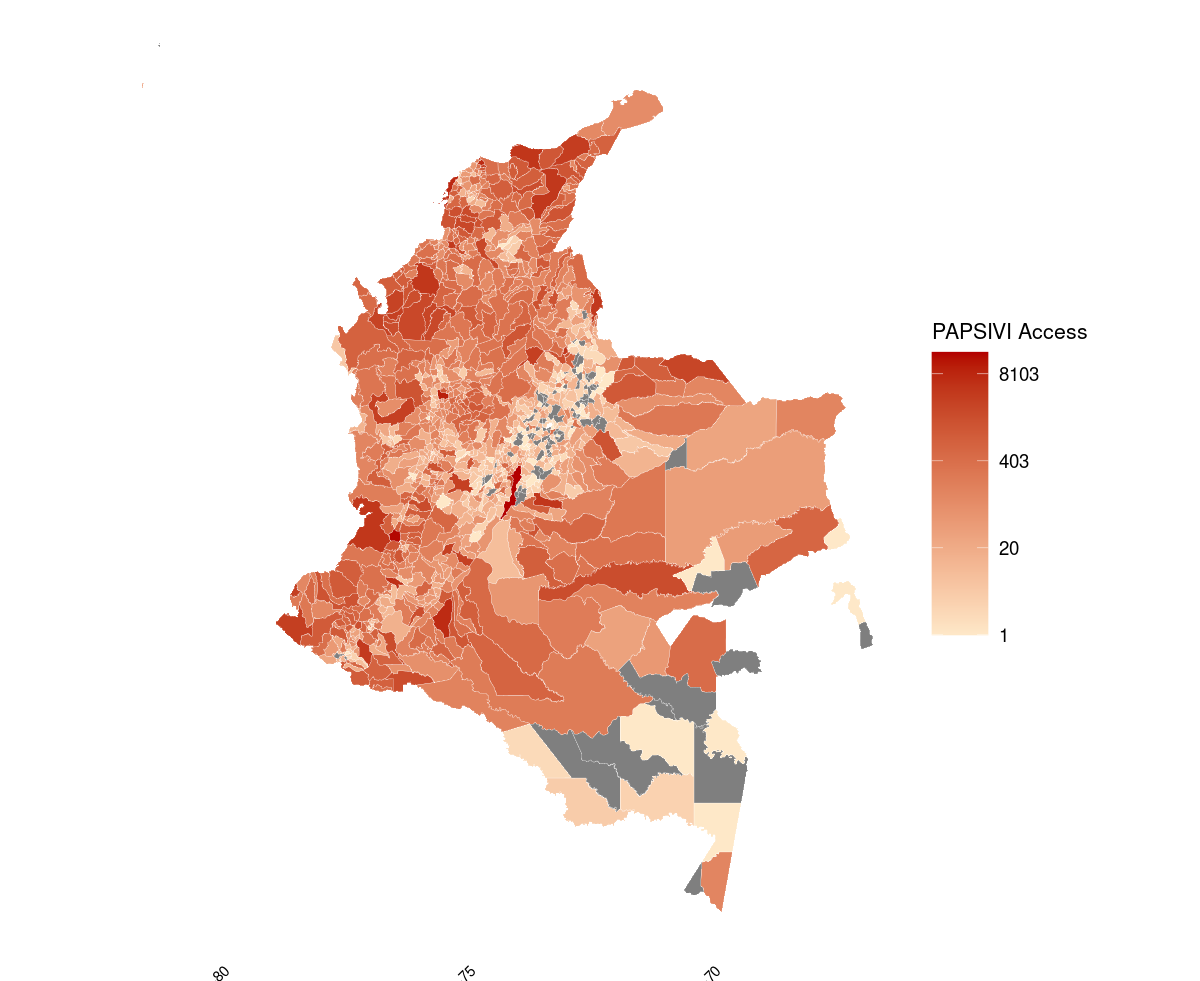


**Tables S1.** Original Spanish language armed conflict exposure categories and translations used in this study

| *Original Spanish language armed conflict exposure category* | *Translation used in this study* |
| --- | --- |
| Abandono o despojo forzado de tierras | Forced land abandonment / dispossession |
| Acto terrorista / atentados / combates / enfrentamientos / hostigamientos | Witness to terrorism or combat |
| Amenaza | Threats |
| Confinamiento | Confinement |
| Delitos contra la libertad y la integridad sexual en desarrollo del conflicto armado | Sexual violence |
| Desaparición forzada | Forced disappearance |
| Desplazamiento forzado | Forced displacement |
| Homicidio | Homicide |
| Lesiones personales físicas | Physical injuries |
| Lesiones personales psicológicas | Psychological injuries |
| Minas antipersonal, munición sin explotar y artefacto explosivo improvisado | Exposure to mines / improvised explosives |
| Perdida de bienes muebles o inmuebles | Loss of personal belongings |
| Secuestro | Kidnapping |
| Tortura | Torture |
| Vinculación de niños niñas y adolescentes a actividades relacionadas con grupos armados | Child recruitment to armed groups |

**Table S2.** Multilevel model odds ratio estimates for associations between armed conflict exposures and access to PAPSIVI including all registrations of armed conflict exposures

|  |  | *Odds ratio (95% CIs)* | | |
| --- | --- | --- | --- | --- |
| *Exposure* | *N* | *Model 1* | *Model 2* | *Model 3* |
| Forced land abandonment / dispossession | 31464 | 3.26 (3.18 - 3.35) | 2.78 (2.70 - 2.85) | 2.60 (2.49 - 2.72) |
| Witness to terrorism or combat | 82845 | 1.05 (1.03 - 1.08) | 1.03 (1.01 - 1.06) | 0.96 (0.93 - 0.99) |
| Threats | 520805 | 0.90 (0.89 - 0.91) | 0.86 (0.85 - 0.87) | 0.88 (0.87 - 0.89) |
| Confinement | 75961 | 0.32 (0.30 - 0.33) | 0.29 (0.28 - 0.31) | 0.33 (0.31 - 0.36) |
| Sexual violence | 33715 | 3.08 (3.00 - 3.16) | 2.53 (2.46 - 2.59) | 2.47 (2.40 - 2.54) |
| Forced disappearance | 160499 | 1.13 (1.11 - 1.15) | 1.04 (1.02 - 1.05) | 1.04 (1.02 - 1.07) |
| Forced displacement | 7540257 | 0.98 (0.97 - 0.99) | 1.05 (1.04 - 1.05) | 1.09 (1.08 - 1.09) |
| Homicide | 919051 | 0.87 (0.86 - 0.88) | 0.85 (0.84 - 0.86) | 0.80 (0.79 - 0.81) |
| Physical injury | 13880 | 0.99 (0.94 - 1.05) | 1.37 (1.29 - 1.45) | 1.40 (1.31 - 1.49) |
| Psychological injury | 13949 | 1.77 (1.68 - 1.86) | 1.60 (1.52 - 1.69) | 1.25 (1.15 - 1.37) |
| Mines, improvised explosives | 11250 | 0.70 (0.65 - 0.75) | 1.49 (1.37 - 1.63) | 1.25 (1.10 - 1.43) |
| Loss of personal belongings | 120168 | 1.11 (1.09 - 1.14) | 0.86 (0.84 - 0.88) | 0.76 (0.74 - 0.78) |
| Kidnapping | 33487 | 0.90 (0.86 - 0.93) | 1.01 (0.97 - 1.05) | 1.03 (0.98 - 1.08) |
| Torture | 9912 | 1.42 (1.34 - 1.50) | 1.46 (1.37 - 1.55) | 1.43 (1.34 - 1.53) |
| Child recruitment to armed groups | 7756 | 0.69 (0.63 - 0.76) | 0.93 (0.84 - 1.02) | 0.76 (0.66 - 0.86) |

*Model 1 = unadjusted association. Model 2 = adjusted for sex, age, healthcare regime, ethnicity. Model 3 = adjusted for sex, age, healthcare regime, ethnicity, CERAC exposure to conflict*

**Table S3.** Multilevel model odds ratio estimates for associations between armed conflict exposures and access to PAPSIVI including only first registration of armed conflict exposures

|  |  | *Odds ratio (95% CIs)* | | |
| --- | --- | --- | --- | --- |
| *Exposure* | *N* | *Model 1* | *Model 2* | *Model 3* |
| Forced land abandonment / dispossession | 13053 | 4.78 (4.58 - 5.00) | 3.67 (3.51 - 3.85) | 3.37 (3.12 - 3.64) |
| Witness to terrorism or combat | 42880 | 1.18 (1.14 - 1.22) | 1.06 (1.02 - 1.10) | 0.98 (0.93 - 1.03) |
| Threats | 234475 | 1.15 (1.13 - 1.17) | 0.99 (0.97 - 1.01) | 0.97 (0.95 - 0.99) |
| Confinement | 46609 | 0.31 (0.29 - 0.33) | 0.26 (0.24 - 0.28) | 0.27 (0.24 - 0.31) |
| Sexual violence | 14421 | 3.94 (3.78 - 4.10) | 2.97 (2.85 - 3.10) | 2.85 (2.73 - 2.98) |
| Forced disappearance | 112395 | 1.04 (1.02 - 1.07) | 0.95 (0.92 - 0.97) | 0.95 (0.92 - 0.98) |
| Forced displacement | 7023767 | 1.01 (1.00 - 1.02) | 1.10 (1.09 - 1.11) | 1.15 (1.14 - 1.17) |
| Homicide | 734064 | 0.74 (0.73 - 0.75) | 0.73 (0.72 - 0.74) | 0.69 (0.68 - 0.70) |
| Physical injury | 5131 | 1.21 (1.09 - 1.34) | 1.70 (1.52 - 1.90) | 1.71 (1.51 - 1.94) |
| Psychological injury | 7830 | 1.81 (1.67 - 1.95) | 1.58 (1.45 - 1.71) | 1.15 (1.00 - 1.32) |
| Mines, improvised explosives | 6461 | 0.61 (0.54 - 0.70) | 1.52 (1.30 - 1.77) | 1.24 (0.96 - 1.59) |
| Loss of personal belongings | 67122 | 1.21 (1.17 - 1.24) | 0.85 (0.82 - 0.88) | 1.01 (0.96 - 1.05) |
| Kidnapping | 19553 | 0.90 (0.85 - 0.96) | 0.93 (0.87 - 0.99) | 0.93 (0.87 - 1.01) |
| Torture | 4196 | 1.58 (1.42 - 1.75) | 1.52 (1.37 - 1.70) | 1.46 (1.29 - 1.65) |
| Child recruitment to armed groups | 5107 | 0.70 (0.61 - 0.80) | 0.89 (0.76 - 1.03) | 0.62 (0.50 - 0.77) |

*Model 1 = unadjusted association. Model 2 = adjusted for sex, age, healthcare regime, ethnicity. Model 3 = adjusted for sex, age, healthcare regime, ethnicity, CERAC exposure to conflict. 95% CIs = 95% confidence intervals.*

**Table S4.** Stratified results for significant interactions with sex. n/c = not calculable

| *Exposure* | *Sex* | *Stratified estimate* | *Interaction p* |
| --- | --- | --- | --- |
| Child recruitment to armed groups | Female | 0.90 (0.76 - 1.07) | 0.00322 |
| Child recruitment to armed groups | Male | 0.61 (0.49 - 0.75) |  |
| Child recruitment to armed groups | Other | n/c |  |
| Confinement | Female | 0.36 (0.33 - 0.40) | 0.00214 |
| Confinement | Male | 0.32 (0.29 - 0.35) |  |
| Confinement | Other | n/c |  |
| Forced displacement | Female | 1.06 (1.05 - 1.07) | <0.00001 |
| Forced displacement | Male | 1.17 (1.15 - 1.18) |  |
| Forced displacement | Other | 0.72 (0.60 - 0.86) |  |
| Forced land abandonment / dispossession | Female | 2.41 (2.26 - 2.57) | 0.00286 |
| Forced land abandonment / dispossession | Male | 2.83 (2.67 - 3.01) |  |
| Forced land abandonment / dispossession | Other | 2.00 (0.19 - 20.93) |  |
| Kidnapping | Female | 1.21 (1.12 - 1.30) | <0.00001 |
| Kidnapping | Male | 0.92 (0.86 - 0.98) |  |
| Kidnapping | Other | 2.09 (0.81 - 5.37) |  |
| Loss of personal belongings | Female | 0.78 (0.76 - 0.81) | 0.00032 |
| Loss of personal belongings | Male | 0.75 (0.72 - 0.78) |  |
| Loss of personal belongings | Other | 0.89 (0.32 - 2.45) |  |
| Physical injury | Female | 1.83 (1.67 - 2.00) | <0.00001 |
| Physical injury | Male | 1.06 (0.95 - 1.17) |  |
| Physical injury | Other | 1.56 (0.73 - 3.36) |  |
| Psychological injury | Female | 1.37 (1.21 - 1.54) | 0.04166 |
| Psychological injury | Male | 1.14 (1.00 - 1.30) |  |
| Psychological injury | Other | 4.32 (0.12 - 157.38) |  |
| Sexual violence | Female | 2.49 (2.42 - 2.56) | 0.00015 |
| Sexual violence | Male | 2.28 (1.96 - 2.65) |  |
| Sexual violence | Other | 1.43 (1.10 - 1.86) |  |
| Threats | Female | 0.92 (0.91 - 0.94) | <0.00001 |
| Threats | Male | 0.80 (0.78 - 0.82) |  |
| Threats | Other | 1.14 (0.92 - 1.40) |  |
| Torture | Female | 1.52 (1.39 - 1.66) | 0.01899 |
| Torture | Male | 1.32 (1.18 - 1.48) |  |
| Torture | Other | 1.24 (0.59 - 2.64) |  |

**Table S5.** Stratified results for significant interactions with healthcare regime.

| *Exposure* | *Regime* | *Stratified estimate* | *Interaction p* |
| --- | --- | --- | --- |
| Child recruitment to armed groups | Contributory | 0.96 (0.76 - 1.22) | 0.00345 |
| Child recruitment to armed groups | Subsidised | 0.69 (0.59 - 0.80) |  |
| Confinement | Contributory | 0.35 (0.29 - 0.42) | <0.00001 |
| Confinement | Subsidised | 0.34 (0.31 - 0.36) |  |
| Forced disappearance | Contributory | 0.98 (0.93 - 1.02) | <0.00001 |
| Forced disappearance | Subsidised | 1.07 (1.04 - 1.09) |  |
| Forced displacement | Contributory | 1.20 (1.18 - 1.22) | <0.00001 |
| Forced displacement | Subsidised | 1.05 (1.04 - 1.06) |  |
| Homicide | Contributory | 0.69 (0.67 - 0.70) | <0.00001 |
| Homicide | Subsidised | 0.85 (0.84 - 0.86) |  |
| Kidnapping | Contributory | 0.83 (0.76 - 0.91) | <0.00001 |
| Kidnapping | Subsidised | 1.17 (1.10 - 1.24) |  |
| Mines, improvised explosives | Contributory | 1.65 (1.28 - 2.14) | 0.00273 |
| Mines, improvised explosives | Subsidised | 1.15 (0.99 - 1.34) |  |
| Psychological injury | Contributory | 0.89 (0.72 - 1.09) | 0.00009 |
| Psychological injury | Subsidised | 1.38 (1.25 - 1.52) |  |
| Sexual violence | Contributory | 3.13 (2.97 - 3.30) | <0.00001 |
| Sexual violence | Subsidised | 2.27 (2.20 - 2.35) |  |
| Threats | Contributory | 0.89 (0.86 - 0.92) | 0.00943 |
| Threats | Subsidised | 0.87 (0.86 - 0.89) |  |
| Witness to terrorism or combat | Contributory | 0.73 (0.69 - 0.78) | 0.00007 |
| Witness to terrorism or combat | Subsidised | 1.02 (0.99 - 1.06) |  |

**Table S6.** Stratified results for significant interactions with ethnic minority status.

| *Exposure* | *Ethnic minority* | *Stratified estimate* | *Interaction p* |
| --- | --- | --- | --- |
| Confinement | No | 0.50 (0.45 - 0.55) | <0.00001 |
| Confinement | Yes | 0.25 (0.23 - 0.28) |  |
| Forced disappearance | No | 1.02 (1.00 - 1.04) | <0.00001 |
| Forced disappearance | Yes | 1.22 (1.16 - 1.30) |  |
| Forced displacement | No | 1.12 (1.11 - 1.13) | <0.00001 |
| Forced displacement | Yes | 0.93 (0.92 - 0.95) |  |
| Forced land abandonment / dispossession | No | 2.59 (2.48 - 2.72) | 0.00344 |
| Forced land abandonment / dispossession | Yes | 2.37 (2.05 - 2.74) |  |
| Homicide | No | 0.77 (0.76 - 0.78) | <0.00001 |
| Homicide | Yes | 1.08 (1.05 - 1.12) |  |
| Kidnapping | No | 1.00 (0.95 - 1.05) | 0.00313 |
| Kidnapping | Yes | 1.31 (1.15 - 1.50) |  |
| Physical injury | No | 1.31 (1.22 - 1.42) | 0.00665 |
| Physical injury | Yes | 1.70 (1.49 - 1.95) |  |
| Sexual violence | No | 2.53 (2.45 - 2.61) | 0.00359 |
| Sexual violence | Yes | 2.30 (2.18 - 2.42) |  |
| Threats | No | 0.85 (0.83 - 0.86) | <0.00001 |
| Threats | Yes | 0.96 (0.93 - 0.99) |  |
| Witness to terrorism or combat | No | 0.93 (0.90 - 0.96) | <0.00001 |
| Witness to terrorism or combat | Yes | 1.04 (0.98 - 1.10) |  |
